# Estimating hunting harvest from partial reporting: a Bayesian approach

**DOI:** 10.1038/s41598-020-77988-x

**Published:** 2020-12-03

**Authors:** Tom Lindström, Göran Bergqvist

**Affiliations:** 1 Department of Physics, Chemistry and Biology, Division of Theoretical Biology, Linköping University, 581 83 Linköping, Sweden; 2 Swedish Association for Hunting and Wildlife Management, Öster Malma, 611 91 Nyköping, Sweden; 3 Southern Swedish Forest Research Centre, Faculty of Forest Sciences, Swedish University of Agricultural Sciences, PO Box 49, 230 53 Alnarp, Sweden

**Keywords:** Ecology, Ecology, Environmental sciences

## Abstract

Quantifying hunting harvest is essential for numerous ecological topics, necessitating reliable estimates. We here propose novel analytical tools for this purpose. Using a hierarchical Bayesian framework, we introduce models for hunting reports that accounts for different structures of the data. Focusing on Swedish harvest reports of red fox (*Vulpes vulpes*), wild boar (*Sus scrofa*), European pine marten (*Martes martes*), and Eurasian beaver (*Castor fiber*), we evaluated predictive performance through training and validation sets as well as Leave One Out Cross Validation. The analyses revealed that to provide reliable harvest estimates, analyses must account for both random variability among hunting teams and the effect of hunting area per team on the harvest rate. Disregarding the former underestimated the uncertainty, especially at finer spatial resolutions (county and hunting management precincts). Disregarding the latter imposed a bias that overestimated total harvest. We also found support for association between average harvest rate and variability, yet the direction of the association varied among species. However, this feature proved less important for predictive purposes. Importantly, the hierarchical Bayesian framework improved previously used point estimates by reducing sensitivity to low reporting and presenting inherent uncertainties.

## Introduction

Game management rely on relevant and cost-efficient monitoring data of sufficient quality and spatial resolution. For many game species, harvest statistics are the only observations available, making them essential to most management programs^1^. Reliable estimates are essential, and there is a demand for new statistical approaches for this purpose.

Harvest reporting systems vary among countries. For instance, reporting is compulsory in many European countries but voluntary in others. Examples of voluntary reporting include the United Kingdom and Cyprus (all species) and Finland (most species)^2^.

In Sweden, harvest reporting is voluntary for most game species but mandatory for species with pre-determined harvest quotas, e.g. moose (*Alces alces*), red deer (*Cervus elaphus*) and large carnivores such as wolf (*Canis lupus*). Harvest of species without pre-determined harvest quotas is regulated by adaptions of the length of the open season, which is decided by the Swedish government based on suggestions from the Swedish Environmental Protection Agency, whereas harvest quotas are decided by the respective County Administrative board. However, the right to hunt and ownership of felled game belongs to the landowner (before it is felled‚ the game belongs to no one). Almost all land suitable for hunting is occupied by hunting teams consisting of landowners and/or hunters leasing hunting rights. Hunting teams are typically geographically stable over time, making it a suitable and stable unit for reporting. Teams annually report harvest and hunting area together with their geographic position to the level of Hunting Management Precinct (HMP, Swedish: Jaktvårdskrets, N = 320 for the hunting year 2015/2016), geographic divisions based on the organisation of The Swedish Association for Hunting and Wildlife Management (SAHWM). Hunting teams are encouraged via information meetings, social media, and magazines to report their harvest to the organisation. In this information, the importance of reporting also if no game was felled (zero reports) is stressed.

The NGO SAHWM has a public commission from the Swedish Government to provide annual harvest estimates for all species with voluntary reporting. Data on harvest is made public online (www.viltdata.se) and is reported annually to the Swedish Government. The current estimation method extrapolates linearly from reported harvest for each HMP, corresponding to the assumption that harvest rate is uniform within each HMP. While straightforward, the system has two major issues. First, it provides only a point estimate without any measure of uncertainty. Second, it is potentially sensitive to low reporting rates, especially if intra-HMP variability among hunting teams is prominent.

At large spatial scales, harvest vary due to e.g. game species composition, game population density, and hunter abundance. At finer spatial scales, harvest variation may be caused by e.g. microhabitat conditions or random variation in game preferences. Anecdotal observations have also indicated that harvest rate might be higher for small hunting areas compared to larger ones.

Bayesian statistics is well suited to analyse and transparently present uncertainties and is used increasingly in ecological research^3^. Because it is straightforward to carry uncertainties forward to the prediction, it is a suitable system to estimate harvest for unreported hunting areas. An appealing feature of Bayesian analysis is the ability to tailor models for the considered system and data. Specifically, Hierarchical Bayesian Modelling (HBM) allows analysts to address multiple levels of uncertainty^4^.

Aiming at both system specific and general insight, the purpose of this study is to introduce a Bayesian framework to improve hunting harvest estimation. We propose a set of Bayesian models and evaluate performance through training and validation sets as well as Pareto Smoothed Importance Sampling Leave One Out Cross Validation (PSIS-LOO-CV)^5^.

## Materials and methods

### Focal species

Currently, there are 49 species for which SAHWM estimates annual harvest across Sweden. We focused on four species that exemplifies game harvested at high (H) or low (L) numbers and uniformly (U) or variably (V) across Sweden: red fox (*Vulpes vulpes*, HU), wild boar (*Sus scrofa*, HV), European pine marten (*Martes martes*, LU), and Eurasian beaver (*Castor fiber*, LV).

Red fox is one of the most abundant small predators in the world^6^ and is common throughout Sweden, ranging from city parks to the high mountains. Red fox is harvested all over Sweden and is hunted for its pelt and to reduce predation on other game species.

The present Swedish wild boar population was established in the 1980s from escapes from enclosures and, likely, deliberate releases. Densities have increased rapidly in recent years, causing great damage to agriculture. Wild boar is abundant in most of southern and central Sweden but absent from the northern parts of the country.

Marten is abundant throughout Sweden. It is an omnivore and the diet consist mainly of smaller animals, but also insects and berries. Marten is hunted for its pelt, often by specialized trappers.

Beaver was once eradicated in Sweden due to its valuable pelt but was reintroduced in the 1920s. It is tied to freshwater and eats bark and branches from hardwood trees. Beaver is present in most of Sweden, except for the southernmost parts. It is hunted for its pelt, often by specialised trappers.

### Data

**Figure 1:**
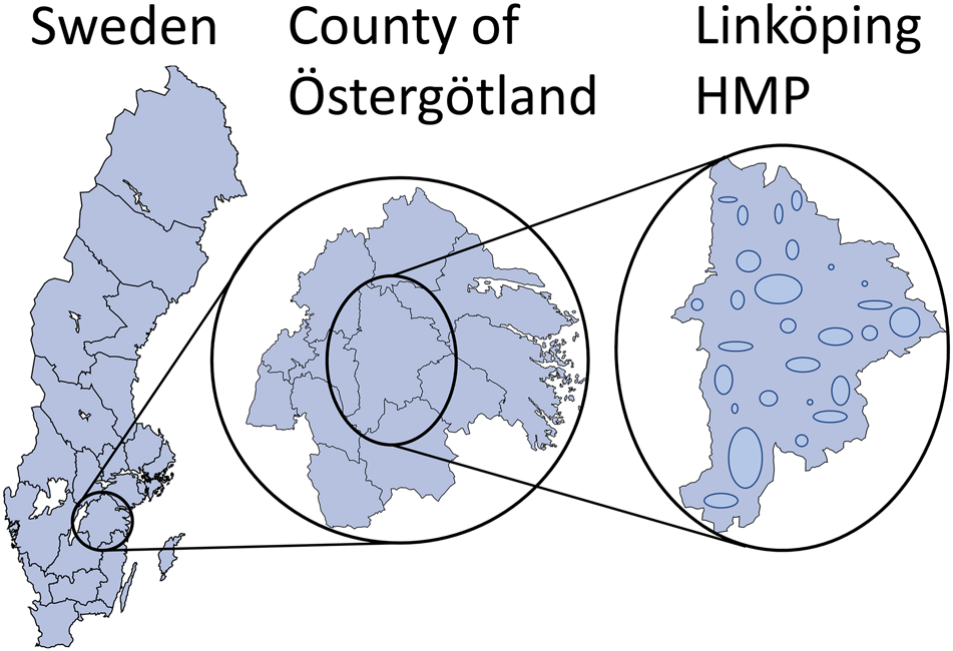
Spatial units of interest. Sweden is divided into counties, which are divided into hunting management precincts (HMPs). The example HMP Linköping in the county Östergötland contained 28 reporting teams, whose locations within their HMP is unknown. The figure was generated in R (version 3.6.3, https://www.r-project.org/) and Microsoft PowerPoint (version 16.0.13231.20110, https://www.microsoft.com/en/microsoft-365/powerpoint).

For each hunting year (July 1–June 30), hunting teams voluntarily report their total harvest to SAHWM, most commonly through the SAHWM-owned database Viltdata (www.viltdata.se), with the opportunity to report their harvest continuously during the hunting year. All reports are checked by local personnel of SAHWM, who, in doubtful cases, contacts the reporting person for clarifications. Examples of inconsistencies that are scrutinized are unusual harvest numbers, no harvest (zero report) in areas where the species in question is common or reported harvest of a species outside its normal distribution area. The reported information includes hunting area, number of individuals harvested for each species, and the HMP in which the hunting ground is located. Thus, HMP is the finest spatial resolution that can be considered from the data. Levels of interest are illustrated in Fig. 1. All individual reports are confidential, and data is only presented at the HMP and higher levels.

Here, we focused on hunting data from 2015/2016, which included 5803 reports. The total reported area of 8,683,074 ha covered 26.5% of the total huntable area, meaning that harvest must be estimated for 73.5% of the huntable area. As wild boar and beaver is not present in all of the country (wild boar not in the north and beaver not in the very south), we excluded counties where all reports indicated zero harvest for the respective species. We also excluded HMPs for which SAHWM do not estimate harvest (north-western HMPs above the tree line and small, urban HMPs in Stockholm and Uppsala). As such, the included HMPs varied among the species.

### Current estimation method

The currently implemented model, henceforth denoted point estimate (P.E.), for hunting harvest in Sweden extrapolates linearly from reports. Denoting with $K_{r,k,l}$ and $A_{r,k,l}$ harvest and hunting team area, respectively, for report *r* in HMP *k* in county *l*, the harvest per area, $\nu_{k,l}$ (animals ha^−1^), is calculated as

1 $$\nu_{k,l} = \left\{ {\begin{array}{*{20}l} {\sum\limits_{{r \in {\mathbf{R}}_{k,l} }} {K_{r,k,l} } /\sum\limits_{{r \in {\mathbf{R}}_{k,l} }} {A_{r,k,l} } \, } \hfill & {{\text{if }}\,{\text{there}}\,{\text{ is}}\,{\text{ at }}\,{\text{least }}\,{\text{one }}\,{\text{report}}\,{\text{ for}}\,{\text{ HMP}}\, \, k,l \, } \hfill \\ {\sum\limits_{{r \in {\mathbf{R}}_{l} }} {K_{r,k,l} } /\sum\limits_{{r \in {\mathbf{R}}_{l} }} {A_{r,k,l} } \, } \hfill & {{\text{if }}\,{\text{there}}\,{\text{ is }}\,{\text{no}}\,{\text{ report }}\,{\text{for}}\,{\text{ HMP }}\,k,l} \hfill \\ \end{array} } \right.,$$

where ${\mathbf{R}}_{k,l}$ is the set of reports for HMP *k* in county *l*, and ${\mathbf{R}}_{l}$ is the set of reports for county *l.* Denoting the total huntable area for HMP *k* in county *l* as $T_{k,l}$ and defining the corresponding unreported area as

2 $$U_{k,l} = T_{k,l} - \sum\limits_{{r \in {\mathbf{R}}_{k,l} }} {A_{k,l} } ,$$

the total estimated HMP harvest is estimated as

3 $$E_{k,l} = \sum\limits_{{r \in {\mathbf{R}}_{k,l} }} {K_{r,k,l} } + \nu_{k,l} U_{k,l} ,$$

i.e. the sum of reported harvest for plus the estimated harvest for the unreported area. The nationwide harvest is given by

4 $$\ddot{E} = \sum\limits_{{l \in {\mathbf{l}}}} {\sum\limits_{{k \in {\mathbf{k}}_{l} }} {E_{k,l} } } .$$

### Proposed method

We propose Bayesian equivalents to the current model. The analysis consists of three steps: analysis of reported hunting areas, analysis of reported harvest, and posterior prediction of unreported harvest volumes.

#### Analysis of reported hunting areas

We modelled the set of reported hunting areas **A** such that the area of report *r* in HMP *k* in county *l* was modelled as

5 $$A_{r,k,l} \sim \text{Log-Normal}\left( {\log \left( {m_{k,l} } \right),s_{l} } \right),$$

where $\log \left( {m_{k,l} } \right)$ and $s_{l}$ are the mean and standard deviation, respectively, of hunting areas on the log-scale. We modelled $m_{k,l}$ as

6 $$m_{k,l} = \exp \left( {W + L_{l} + C_{k,l} } \right),$$

where $W$, $L_{l}$, and $C_{k,l}$ are the nationwide, county, and HMP level effects, respectively. We further modelled.

7 $$C_{k,l} \sim \text{Normal}\left( {0,t} \right),$$

where *t* is the standard deviation of HMP level effects and modelled as

8 $$t \sim \text{Log-Normal}\left( {\Omega_{t} ,S_{t} } \right).$$

Similarly, county effects were modelled as

9 $$L_{l} \sim \text{Normal}\left( {0,T} \right),$$

where *T* is the standard deviation of county level effects and modelled with prior

10 $$T \sim \text{Log-Normal}\left( {\Omega_{T} ,S_{T} } \right).$$

We expressed the prior for the nationwide effect as

11 $$W \sim \text{Normal}\left( {\Omega_{W} ,S_{W} } \right).$$

Because the model is expressed on the log-scale, $s_{l}$ in Eq. (5) is a scale free measure of variability in hunting area within each HMP. Exploratory analysis of the data suggested within HMP variability in hunting area per team—whether measured by variance or coefficient of variation—differed among counties. To account for this pattern, we modelled $s_{l}$ as

12 $$s_{l} = \exp \left( {u + v_{l} } \right),$$

where *u* and $v_{l}$ are interpreted as the nationwide and county level effects, respectively, on the log-transform of this variability. County level effects on variability were modelled as

13 $$v_{l} \sim \text{Normal}\left( {0,z} \right),$$

where

14 $$z \sim \text{Log-Normal}\left( {\Omega_{z} ,S_{z} } \right).$$

The prior for *u* was modelled as

15 $$u \sim \text{Normal}\left( {\Omega_{u} ,S_{u} } \right).$$

#### Bayesian baseline model for reported harvest

As a first step towards a Bayesian model for harvest data, we assumed hunting within each HMP occurs uniformly with HMP specific rate (animals ha^−1^ yr^−1^), $\mu_{k,l}$, yielding the likelihood for the set of reports ${\mathbf{R}}_{k,l}$

16 $$P\left( {{\mathbf{R}}_{k,l} |\mu_{k,l} } \right) = \prod\limits_{{r \in {\mathbf{R}}_{k,l} }} {{\text{Poiss}} \left( {K_{r,k,l} |\mu_{k,l} A_{r,k,l} } \right)} = {\text{Poiss}} \left( {\sum\limits_{{r \in {\mathbf{R}}_{k,l} }} {K_{r,k,l} } |\mu_{k,l} \sum\limits_{{r \in {\mathbf{R}}_{k,l} }} {A_{r,k,l} } } \right).$$

Solving

17 $$\frac{{d\log \left( {P\left( {{\mathbf{R}}_{k,l} |\mu_{k,l} } \right)} \right)}}{{d\mu_{k,l} }} = \frac{1}{{\mu_{k,l} }}\sum\limits_{{r \in {\mathbf{R}}_{k,l} }} {K_{r,k,l} } - \sum\limits_{{i \in {\mathbf{R}}_{k,l} }} {A_{r,k,l} } = 0,$$

the maximum likelihood (ML) estimate for HMPs with at least one report is

18 $$\hat{\mu }_{k,l} = \sum\limits_{{r \in {\mathbf{R}}_{k,l} }} {K_{r,k,l} } /\sum\limits_{{r \in {\mathbf{R}}_{k,l} }} {A_{r,k,l} }$$

Thus, the Poisson model is an obvious likelihood equivalent to Eq. (1). Using a conjugate gamma prior, a posterior distribution for a Bayesian equivalent can be obtained as

19 $$\begin{aligned} P\left( {\mu_{k,l} |{\mathbf{R}}_{k,l} } \right) & \propto {\text{Poiss}} \left( {\sum\limits_{{r \in {\mathbf{R}}_{k,l} }} {K_{r,k,l} } |\mu_{k,l} \sum\limits_{{r \in {\mathbf{R}}_{k,l} }} {A_{r,k,l} } } \right){\text{Gamma}} \left( {\mu_{k,l} |a,b} \right) \\ & \propto {\text{Gamma}} \left( {\mu_{k,l} |a + \sum\limits_{{r \in {\mathbf{R}}_{k,l} }} {K_{r,k,l} } ,b + \sum\limits_{{r \in {\mathbf{R}}_{k,l} }} {A_{r,k,l} } } \right), \\ \end{aligned}$$

where *a* and *b* are shape and rate parameters, respectively. However, the common approach to elicit vague priors as $a = b =$ “something small” fails for the focal system. Often, $\sum\nolimits_{{r \in {\mathbf{R}}_{k,l} }} {K_{r,k,l} = 0}$, making the posterior of Eq. (19) sensitive to $a$. Also, occasional HMP have no reports ($\sum\nolimits_{{r \in {\mathbf{R}}_{k,l} }} {A_{r,k,l} = 0}$), making the posterior sensitive to $b$. Thus, when reporting and/or harvest is low, it would be useful to borrow strength from the harvest pattern in surrounding HMPs. We defined

20 $$\log \left( {\mu_{k,l} } \right) = \chi_{k,l} + \lambda_{l} + \omega ,$$

where $\chi_{k,l}$, $\lambda_{l}$ and $\omega$ are HMP, county and nationwide effects, respectively. This promotes a straightforward hierarchical structure for HMP and county effects as

21 $$\begin{aligned} \chi_{k,l} & \sim {\text{Normal}} \left( {0,\sigma } \right) \\ \lambda_{l} & \sim {\text{Normal}} \left( {0,\tau } \right), \\ \end{aligned}$$

where $\sigma$ and $\tau$ are standard deviations of HMP and county effects, respectively, and assigned priors

22 $$\begin{aligned} \sigma & \sim \text{Log-Normal}\left( {\Omega_{\sigma } ,S_{\sigma } } \right) \\ \tau & \sim \text{Log-Normal}\left( {\Omega_{\tau } ,S_{\tau } } \right). \\ \end{aligned}$$

We refer to this Bayesian baseline model for harvest data as ${\rm M}_{0}$.

##### Within HMP variation

The assumption of ${\rm M}_{0}$ that all hunting teams within an HMP harvests with equal rate $\mu_{k,l}$ may be invalid. While underlying reasons for intra-HMP variability are for most part intractable from the available data, non-homogeneous harvesting should be accounted for if it improves prediction. Thus, we specified an alternative likelihood

23 $$\begin{aligned} & P\left( {{\mathbf{R}}_{k,l} |\mu_{k,l} ,\alpha_{k,l} } \right) \\ & \quad = \prod\limits_{{r \in {\mathbf{R}}_{k,l} }} {\left[ {\frac{{\left( {{{\alpha_{k,l} } \mathord{\left/ {\vphantom {{\alpha_{k,l} } {\left( {\mu_{k,l} A_{r,k,l} } \right)}}} \right. \kern-\nulldelimiterspace} {\left( {\mu_{k,l} A_{r,k,l} } \right)}}} \right)^{{\alpha_{k,l} }} }}{{\Gamma \left( {K_{r,k,l} + 1} \right)\Gamma \left( {K_{r,k,l} + \alpha_{k,l} } \right)}}\frac{{\Gamma \left( {K_{r,k,l} + \alpha_{k,l} } \right)}}{{\left( {{{\alpha_{k,l} } \mathord{\left/ {\vphantom {{\alpha_{k,l} } {\left( {\mu_{k,l} A_{r,k,l} } \right)}}} \right. \kern-\nulldelimiterspace} {\left( {\mu_{k,l} A_{r,k,l} } \right)}} + 1} \right)^{{K_{r,k,l} + \alpha_{k,l} }} }}} \right],} \\ \end{aligned}$$

which is the Gamma–Poisson mixture distribution parameterized from its mean $\mu_{k,l} A_{r,k,l}$ and shape parameter $\alpha_{k,l}$. This corresponds to the assumption that hunting follows a Poisson process with a team specific rate, which is assumed to be gamma distributed. We keep the notation $\mu_{k,l}$ as in Eq. (16) because the Poisson distribution is the limiting case of the Gamma–Poisson mixture as $\alpha_{k,l} \to \infty$, and for finite $\alpha_{k,l}$, $\mu_{k,l}$ is the average harvest rate for teams in circuit *k* in county *l*. The Gamma–Poisson mixture can be reparametrized as the Negative Binomial (NB), yet we use the Gamma–Poisson notation to avoid confusion with the standard parameterization of the NB.

We considered models for $\alpha_{k,l}$ that either includes or excludes association between average harvest rate $\mu_{k,l}$ and intra-HMP variation as

24 $$\log \left( {\alpha_{k,l} } \right) = \left\{ {\begin{array}{*{20}l} {\upsilon + \gamma \left( {\chi_{k,l} + \lambda_{l} } \right)} \hfill & {{\text{if}}\,{\text{ including}}\,{\text{rate--variability }}\,{\text{association}}} \hfill \\ \upsilon \hfill & {{\text{if }}\,{\text{excluding }}\,{\text{rate--variability }}\,{\text{association}}} \hfill \\ \end{array} } \right.,$$

where $\gamma > 0$ indicates a positive effect of increased harvest rate (relative to nationwide average $\omega$) on the shape parameter $\alpha_{k,l}$. Large values of $\alpha_{k,l}$ indicate less variability.

We implemented priors

25 $$\gamma \sim \text{Normal}\left( {\Omega_{\gamma } ,S_{\gamma } } \right)$$

26 $$\upsilon \sim \text{Normal}\left( {\Omega_{\upsilon } ,S_{\upsilon } } \right)$$

and used the same hierarchical structure specified for $\mu_{k,l}$ in ${\text{M}}_{0}$.

##### Effect of hunting area on harvest rate

We incorporated an additional parameter $\phi$, modelling the potential effect of a team’s hunting area on its harvest rate per area, extending Eqs. (16) and (23) to

27 $$P\left( {{\mathbf{R}}_{k,l} |\mu_{k,l} ,\phi_{r,k,l} } \right) = \prod\limits_{{r \in {\mathbf{R}}_{k,l}}} {{\text{Poiss}} \left( {K_{r} |\mu_{k,l} A_{r} \delta_{r,k,l} } \right)}$$

and

28 $$\begin{aligned} & P\left( {{\mathbf{R}}_{k,l} |\mu_{k,l} ,\alpha_{k,l} ,\phi_{r,k,l} } \right) \\ & \quad = \prod\limits_{{r \in {\mathbf{R}}_{k,l} }} {\left[ {\frac{{\left( {{{\alpha_{k,l} } \mathord{\left/ {\vphantom {{\alpha_{k,l} } {\left( {\delta_{r,k,l} \mu_{k,l} A_{r,k,l} } \right)}}} \right. \kern-\nulldelimiterspace} {\left( {\delta_{r,k,l} \mu_{k,l} A_{r,k,l} } \right)}}} \right)^{{\alpha_{k,l} }} }}{{\Gamma \left( {K_{r,k,l} + 1} \right)\Gamma \left( {K_{r,k,l} + \alpha_{k,l} } \right)}}\frac{{\Gamma \left( {K_{r,k,l} + \alpha_{k,l} } \right)}}{{\left( {{{\alpha_{k,l} } \mathord{\left/ {\vphantom {{\alpha_{k,l} } {\left( {\delta_{r,k,l} \mu_{k,l} A_{r,k,l} } \right)}}} \right. \kern-\nulldelimiterspace} {\left( {\delta_{r,k,l} \mu_{k,l} A_{r,k,l} } \right)}} + 1} \right)^{{K_{r,k,l} + \alpha_{k,l} }} }}} \right]} , \\ \end{aligned}$$

respectively, where

29 $$\delta_{r,k,l} = \exp \left( {\phi \log \left( {{{A_{r,k,l} } \mathord{\left/ {\vphantom {{A_{r,k,l} } {\overline{m}_{k,l} }}} \right. \kern-\nulldelimiterspace} {\overline{m}_{k,l} }}} \right)} \right).$$

Using ${{A_{r,k,l} } \mathord{\left/ {\vphantom {{A_{r,k,l} } {\overline{m}_{k,l} }}} \right. \kern-\nulldelimiterspace} {\overline{m}_{k,l} }},$ where $\overline{m}_{k,l}$ is the typical hunting team area in the focal HMP, facilitates the interpretation of $\phi$ as the effect of relative hunting area and avoids confusing $\phi$ with large-scale differences in average hunting area among HMPs. To avoid refitting of the hunting area model for each species within each model of harvesting, we defined $\overline{m}_{k,l}$ as the median of the posterior of $m_{k,l}$ defined in Eq. (6) For $\phi = 0$, there is no effect of hunting area. For $\phi < 0$, per area harvest rate decreases with hunting area. We specified the prior

30 $$\phi \sim \text{Normal}\left( {\Omega_{\phi } ,S_{\phi } } \right).$$

#### Model notation

We denote with subscripts $\alpha$, $\gamma$, and/or $\phi$ models that include intra-HMP variability, association between harvest rate and intra-HMP variability, and/or effect of hunting area on harvest rate. Including $\gamma$ is only relevant for models including $\alpha$. Thus, there are six considered Bayesian models: ${\rm M}_{\alpha \gamma \phi }$, ${\rm M}_{\alpha \phi }$, ${\rm M}_{\phi }$, ${\text{\rm M}}_{\alpha \gamma }$,${\rm M}_{\alpha }$, and ${\rm M}_{0}$.

#### Prior elicitation

Our approach was to specify ranges of parameter values that we deemed plausible across species. Defining this range as the 95% central interval of the prior distribution, we allowed for posterior estimates outside of the range, should the likelihood strongly contradict our beliefs. The choice of Normal and Log-Normal functional forms as priors were implemented because these distribution are suitable to incorporate (if however vague) prior beliefs, and we used the rule-of-thumb that ~ 95% of the density lies within two standard deviations of the mean (accounting for the log-transform when using the Log-Normal).

Table 1 lists prior parameters and the ranges from which they were derived below.

**Table 1:** List of parameters, their definition, and (for highest level parameters) elicited ranges describing 95% prior probability and corresponding hyperparameters defining the prior distributions.

Parameter	Definition	95% Prior range	Prior parameters
**Hunting area parameters**			
$W$	Nationwide average log-area per hunting team	[2.3, 9.2]	$\Omega_{W} = 5.8$, $S_{W} = 1.7$
$L_{l}$	County level effect for county *l*		
$C_{k,l}$	HMP level effect for HMP *k* in county *l*		
*T*	Standard deviation of county level effects	[0.20, 2.3]	$\Omega_{T} = - 0.38$, $S_{T} = 0.61$
*t*	Standard deviation of HMP level effects	[− 1.3, 2.3]	$\Omega_{t} = - 1.3$, $S_{t} = 0.86$
*u*	Nationwide average intra-HMP variation (log standard deviation) in log area per team	[− 2.3, 0.77]	$\Omega_{u} = - 0.77$, $S_{u} = 0.36$
$v_{l}$	County level effect on intra-HMP variation (log standard deviation) in log area per team in county *l*		
$s_{l}$	$s_{l} = \exp \left( {u + v_{l} } \right)$		
$z$	Standard deviation of county level effect on intra-HMP variation	[0.095, 2.3]	$\Omega_{z} = - 1.5$, $S_{z} = 0.80$
**Hunting rate parameters**			
$\mu_{k,l}$	Average hunting rate per team in HMP *k* in county *l*		
$\omega$	Nationwide mean log hunting rate per team	[− 17, 0]	$\Omega_{\omega } = - 8.7$, $S_{\omega } = 4.3$
$\lambda_{l}$	County level effect on log hunting rate for county *l*		
$\chi_{k,l}$	HMP level effect on log hunting rate for HMP *k* in county *l*		
$\tau$	Standard deviation of county level effects on log hunting rate	[0.20, 2.3]	$\Omega_{\tau } = - 0.38$, $S_{\tau } = 0.61$
$\sigma$	Standard deviation of HMP level effects on log hunting rate	[− 1.3, 2.3]	$\Omega_{\sigma } = - 1.3$, $S_{\sigma } = 0.86$
$\alpha_{k,l}$	Shape parameter for intra-HMP variation in hunting rate for		
$\upsilon$	Average log shape parameter for intra-HMP variation in hunting rate	[− 6.0, 6.0]	$\Omega_{\upsilon } = 0$, $S_{\upsilon } = 3.0$
$\gamma$	Effect of HMP specific average hunting rate per team on log shape parameter for intra-HMP variation in hunting rate	[− 2.0, 2.0]	$\Omega_{\gamma } = 0$, $S_{\gamma } = 1.0$
$\phi$	Effect of hunting team area on harvest rate per area	[− 1.0, 1.0]	$\Omega_{\phi } = 0$, $S_{\phi } = 0.50$

The highest-level parameters for the hunting area model are *W*, *T*, *t*, *u*, and *z*. The nationwide effect on average hunting area, *W*, is expressed for log-area, and we specified the plausible range as [2.3, 9.2], corresponding to the vague prior belief that the typical hunting area per team is (with 95% certainty) between 10 and 10,000 ha.

A plausible range for the standard deviation of county effects on hunting area, *T*, can be derived from expectations about relative difference among counties. We knew there are geographic differences and derived a lower range from the assumption that hunting areas in a county two standard deviations above or below the geometric average differ from the average by at least a factor of 1.5. Conversely, we derived the upper range from the assumption that hunting areas in a county that is two standard deviations from the geometric average unlikely differs by more than a factor of 100 from the average. The corresponding range on the log-scale used to parameterize the Log-Normal distribution is [0.20, 2.3]. We derived the prior of the standard deviation of county effects, *t*, from similar expectations, yet with the lower and upper range at a factor of 1.1 and 20, respectively. The corresponding range on the log-scale is [− 1.3, 2.3].

Parameter *u* models the nationwide effect on $s_{l}$, the standard deviation of intra-HMP variability in hunting area per team. Expressing prior beliefs about this distribution is more intuitive when considering its coefficient of variation, $c = \sqrt {e^{{s_{l}^{2} }} - 1}$. We specified high and low variation as $c = 10$ (standard deviation of hunting areas within a HMP is ten times the average) and $c = 0.1$ (standard deviation of hunting areas within an HMP is 10% of the average). This correspond to a range for *u* as [− 2.3, 0.77]. Parameter *z* models variability among counties in terms of intra-HMP variability. We expressed a plausible range based on relative difference between 0.1 (little variation among counties) and 10 (high variation between counties), which corresponds to a range for *z* between [0.095, 2.3].

The harvest rate models include up to six highest-level parameters: $\omega$, $\tau$, $\sigma$, $\upsilon$, $\gamma$, and $\phi$. We derived the lower limit of the plausible range for $\omega$ from the expectation that the geometric mean harvest rate corresponds to at least one individual across Sweden’s approximately 32,800,000 ha of huntable land. We specified the upper limit from the expectation that the geometric mean rate is unlikely higher than one animal per ha. The corresponding range for $\omega$, which is expressed on the log-scale, is [− 17, 0].

Similar to *T* and *t*, we derived plausible ranges for $\tau$ and $\sigma$ from how effect sizes translates into relative difference among counties and HMPs, respectively. For variability of county effects $\tau$, we assumed a plausible effect size at two standard deviations from the geometric average between a factor of 1.5 and 100, corresponding to a prior range for $\tau$ of [0.20, 2.3]. For $\sigma$, modelling variability of HMPs within a county, we specified plausible relative differences as between a factor of 1.1 and 20, which corresponds to a prior range of [− 1.3, 2.3].

For $\upsilon$, the intercept of $\log \left( {\alpha_{k,l} } \right)$, we utilized the relationship between the Gamma–Poisson mixture’s shape parameter and the coefficient of variation of harvest rates described by the associated Gamma distribution, $c = {1 \mathord{\left/ {\vphantom {1 {\sqrt \alpha }}} \right. \kern-\nulldelimiterspace} {\sqrt \alpha }}$. Under high intra-HMP variability, we assumed *c* may be as high as 20. If variability is low, a plausible *c* could be as low as 1/20. These upper and lower limits for *c* translate to a range of the shape parameter within 0.025 and 400, respectively, which when log-transformed to the scale of $\upsilon$ is [− 6.0, 6.0]. We further assumed $\gamma$ should (with 95% certainty) be limited to an increase or decrease of coefficient of variation with a factor of two with every doubling of the average harvest rate. This corresponds to a prior range for $\gamma$ as [−2.0, 2.0].

If $\phi = - 1$, there is no change in the harvest rate per team with hunting area. If $\phi = 1$, the harvest rate per team grows quadratically with hunting area. Thus, we let these extremes define the prior range for $\phi$.

### Prediction

We performed posterior predictive sampling of harvest for the unreported area by repeatedly sampling team areas and team specific harvest rates according to each candidate model for harvesting. The number of non-reporting hunting teams is unknown, but our framework permits us to sample recursively until we overshoot $U_{k,l}$. To ensure a consistent total area, we truncated the overshooting sample.

Team level harvests are assumed to follow a Poisson process for all models, and we sampled

31 $$K_{k,l}^{(q,h)} \sim {\text{Poiss}} \left( {\theta_{k,l}^{(q,h)} } \right),$$

where $\theta_{k,l}^{(q,h)}$ is the harvest rate for sampled team *h* for posterior sample *q* and is assigned as

32 $$\begin{aligned} \theta_{k,l}^{(q,h)} & = A_{h} \mu_{k,l}^{(q)} \, \,{\text{for }}\,{\rm M}_{0} \\ \theta_{k,l}^{(q,h)} & \sim \text{Gamma}\left( {\alpha_{k,l}^{(q)} ,{{\mu_{k,l}^{(q)} A_{h} } \mathord{\left/ {\vphantom {{\mu_{k,l}^{(q)} A_{h} } {\alpha_{k,l}^{(q)} }}} \right. \kern-\nulldelimiterspace} {\alpha_{k,l}^{(q)} }}} \right) \, \,{\text{for}}\, \, {\rm M}_{\alpha \gamma } \, \,{\text{and }}\,{\rm M}_{\alpha } \\ \theta_{k,l}^{(q,h)} & = \mu_{k,l}^{(q)} A_{h} e^{{\log \left( {\overline{A}_{r} } \right)\phi^{(q)} }} \, \,{\text{for }}\,{\rm M}_{\phi } \\ \theta_{k,l}^{(q,h)} & \sim \text{Gamma}\left( {\alpha_{k,l}^{(q)} ,{{\mu_{k,l}^{(q)} A_{h} e^{{\log \left( {\overline{A}_{h} } \right)\phi^{(q)} }} } \mathord{\left/ {\vphantom {{\mu_{k,l}^{(q)} A_{h} e^{{\log \left( {\overline{A}_{h} } \right)\phi^{(q)} }} } {\alpha_{k,l}^{(q)} }}} \right. \kern-\nulldelimiterspace} {\alpha_{k,l}^{(q)} }}} \right){\text{ for }}\,{\rm M}_{\alpha \gamma \phi } \, \,{\text{and}}\, \, {\rm M}_{\alpha \phi } . \\ \end{aligned}$$

The sampled harvest for the unreported area for sample *q* in HMP *k* in county *l* is given by

33 $$\kappa_{k,l}^{(q)} = \sum\limits_{h = 1}^{{H_{q,k,l} }} {K_{k,l}^{(q,h)} } ,$$

where $H_{q,k,l}$ is the number of sampled teams for posterior draw *q.* The corresponding county and nationwide level samples are

34 $$\dot{\kappa }_{l}^{(q)} = \sum\limits_{{k \in {\mathbf{k}}_{l} }} {\kappa_{k,l}^{(q)} } ,$$

and

35 $$\ddot{\kappa }^{(q)} = \sum\limits_{{l \in {\mathbf{l}}_{{}} }} {\dot{\kappa }_{l}^{(q)} } ,$$

respectively. For each model and species, we simulated 1,000,000 samples, each parameterized with a random combination of samples from Markov Chain Monte Carlo (MCMC) analysis of hunting area and harvest rates.

### Computation

All analyses were executed in R^7^. Bayesian analyses were implemented with Stan^8^, using the ‘RStan’ package^9^. Stan’s Hamiltonian Monte Carlo algorithm facilitates efficient sampling and often outperforms other samplers in terms of computation time^10^. Tuning parameters were kept at Stan’s defaults, except for the targeted long-term proposal acceptance probability (adapt_delta), which was set to 0.99 due to occasional warnings of divergent transitions in preliminary analyses.

To circumvent poor mixing due to funnel-like distributions, we introduced as primitive parameters

36 $$\begin{aligned} \lambda_{l}^{*} & \sim \text{Normal}\left( {0,1} \right) \\ \chi_{k,l}^{*} & \sim \text{Normal}\left( {0,1} \right). \\ \end{aligned}$$

and implemented the transforms

37 $$\begin{aligned} \lambda_{l} & = \sigma \lambda_{l}^{*} \\ \chi_{k,l} & = \tau \chi_{k,l}^{*} . \\ \end{aligned}$$

Unreasonable seeding conditions for the MCMC may cause numerical issues, and we specified for each chain in the harvest analysis seeds as random draws

38 $$\begin{aligned} \omega^{{\left( {seed} \right)}} & \sim \text{Normal}\left( {\frac{{\sum\limits_{{l \in {\mathbf{H}}}} {\log \left( {\sum\limits_{{r \in {\mathbf{R}}_{l} }} {K_{r,k,l} } /\sum\limits_{{r \in {\mathbf{R}}_{l} }} {A_{r,k,l} } } \right)} }}{{|{\mathbf{H}}|}},1} \right) \\ \lambda_{l}^{{\left( {seed} \right)}} & \sim \text{Normal}\left( {\log \left( {\sum\limits_{{r \in {\mathbf{R}}_{l} }} {K_{r,k,l} } /\sum\limits_{{r \in {\mathbf{R}}_{l} }} {A_{r,k,l} } } \right) - \omega^{{\left( {seed} \right)}} ,0.5} \right) \\ \chi_{k,l}^{{\left( {seed} \right)}} & \sim \left\{ {\begin{array}{*{20}l} {\text{Normal}\left( {{\text{l}}og\left( {\sum\limits_{{r \in {\mathbf{R}}_{k,l} }} {K_{r,k,l} } /\sum\limits_{{r \in {\mathbf{R}}_{k,l} }} {A_{r,k,l} } } \right) - \lambda_{l}^{{\left( {seed} \right)}} - \omega^{{\left( {seed} \right)}} ,0.5} \right)} \hfill & {{\text{if}}\, \, \sum\limits_{{r \in {\mathbf{R}}_{k,l} }} {K_{r,k,l} } > 0} \hfill \\ {\text{Normal}\left( {0,0.5} \right)} \hfill & {{\text{if}}\, \, \sum\limits_{{r \in {\mathbf{R}}_{k,l} }} {K_{r,k,l} } = 0} \hfill \\ \end{array} } \right. \\ \gamma^{(seed)} & \sim \text{Normal}\left( {0,1} \right) \\ \tau^{(seed)} & \sim {\text{Gamma}}\left( {5,5} \right) \\ \sigma^{(seed)} & \sim {\text{Gamma}}\left( {5,5} \right) \\ \phi^{(seed)} & \sim \text{Normal}\left( {0,1} \right) \\ \upsilon^{(seed)} & \sim \text{Normal}\left( {0,1} \right). \\ \end{aligned}$$

These seeding conditions were picked somewhat ad hoc to ensure over-dispersed seeding, yet without causing numerical issues or exceptionally long convergence periods.

Under these seeding conditions, transforms, and tuning parameters, no warnings occurred that would indicate unreliable integration, except for one analysis of pine marten with ${\rm M}_{\alpha \phi }$, where one chain got stuck in a local optimum. This was solved by reseeding with a different random draw from Eq. (38). The area analysis encountered no issues when using Stan’s default seeding methods. We ran four chains of 30,000 iterations, including 5000 iteration warmup, and 80% thinning to avoid large output files.

To facilitate faster posterior predictive sampling, we used NIMBLE^11^. This flexible package for Bayesian computation includes the possibility to compile R functions into C++.

### Model comparison and validation

Two methods of validation and model selection were implemented. First, we divided the reports into validation and training sets and evaluated model performance based on their ability to predict the harvest observed in the former after parameterization from the latter. Second, we used cross validation to evaluate the importance of parameters at the level of reports.

#### Training and validation sets

The most relevant scales to evaluate predictive performance is at the levels at which we want to predict, here HMPs, counties, and nation. Unfortunately, we do not have full coverage at any of these levels—therefore the introduced models are needed—which prevents comparison. Instead, we used half the reports (training set) to estimate parameters, performed prediction of unobserved harvest for the area covered by the other half (validation set), and compared performance by the models’ relative probability to capture the harvest in the validation set.

We randomly assigned each report to the training or the validation set. Parameterization was performed according to the "Proposed method" section, yet including only the training data. Prediction was performed according to the "Prediction" section, yet with $U_{k,l}$ set to the area covered by the validation set. The posterior predictive mass (PPM) at the observed harvest was used to quantify predictive performance.

With 1,000,000 samples, the posterior predictive sampling provides a representative description of the predictive distribution. However, the PPM at the observed harvest may be represented by a small number of samples when the predictive distribution includes a wide range of discrete values. We determined PPM based on 10,000 samples at the observed harvest as a cut-off under which we considered sampling randomness could influence results. In these instances, we used jittering kernel density estimation, which offers unbiased kernel estimation for discrete data^12^. Kernel estimation was implemented with the R-package ‘kde1d’^13^,

#### Leave one out cross validation

To further investigate the importance of model features, we performed Leave One Out Cross Validation (LOO-CV). The rationale for the approach is straightforward—one observation (here the harvest per report) at the time is excluded from the analysis, predictive performance is compared across models based on expected log-pointwise density (ELPD), which is the sum over log-predictive density for each excluded observation.

Exact LOO-CV would require re-running the MCMC for each excluded observation, here ~ 5000 times per species and model. Fortunately, Pareto Smoothed Importance Sampling (PSIS) can be used to approximate out-of-sample predictive density from within-sample analysis^5^. The PSIS-LOO-CV framework identifies observations where the approximation is unreliable. Based on recommendations from Vehtari et al.^14^, exact LOO-CV was performed for observations where the Pareto shape parameter exceeded 0.7.

What constitutes a large enough ELPD difference to draw conclusions from depends on the standard errors (SE) of differences in log-predictive density across observations. A difference of two SE would suffice if the focal sample is well-behaved, but a more conservative difference of four SE is considered a safer range to ensure that ELPD differences are valid outside of the focal sample.

## Results

**Figure 2:**
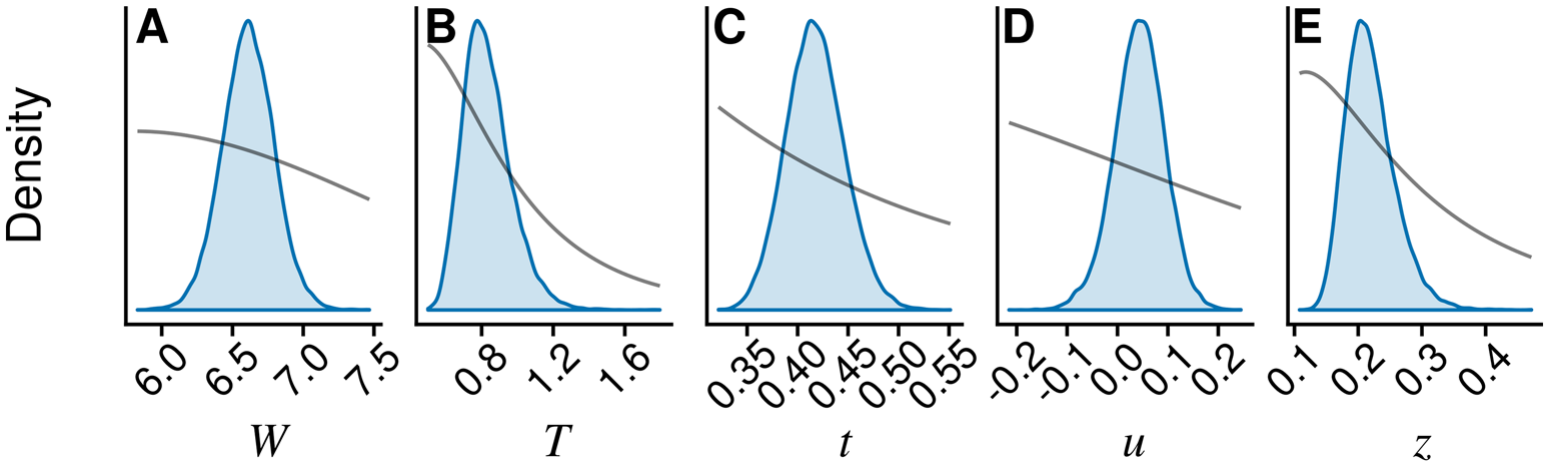
Marginal posterior estimates (shaded densities) of highest-level parameters in the hunting area model. Grey curves are proportional to the implemented prior distributions. The figure was generated in R (version 3.6.3, https://www.r-project.org/).

**Figure 3:**
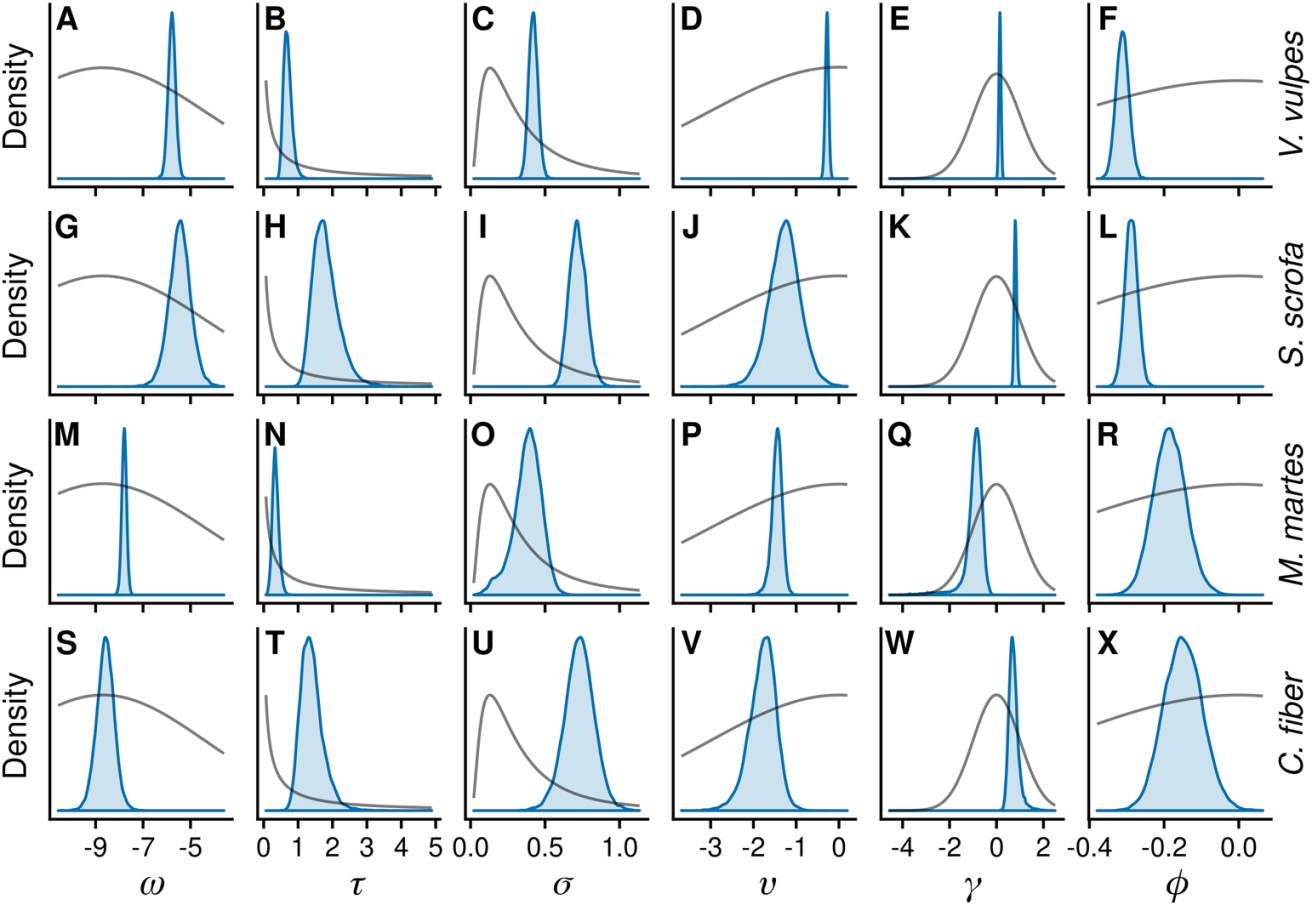
Marginal posterior estimates (filled densities) of highest-level parameters of model ${\rm M}_{\alpha \gamma \phi }$ used for analyses of harvest rates of red fox (*Vulpes vulpes*), wild boar (*Sus scrofa*), pine marten (*Martes martes*), and beaver (*Castor fiber*). Grey curves are proportional to the implemented priors. The figure was generated in R (version 3.6.3, https://www.r-project.org/).

All marginal posterior distributions in Figs. 2 and 3 are notably different from their priors, indicating little prior sensitivity.

### Parameter estimates, area analysis

The standard deviation of county effects *T* and circuit effects *t* were estimated at 0.83 [0.61, 1.10] and 0.42 [0.36, 0.48], respectively, indicating that variability in hunting area per team was larger among counties than among HMPs within counties. The posterior density of standard deviation of county level effect on intra-HMP variation, $z$ (0.22 [0.16, 0.31]), is clearly separated from zero, indicating a pronounced difference among counties in terms of intra-HMP variability.

### Parameter estimates, harvest analysis

Because all reduced models are special or limiting cases of the full model, we focus on ${\rm M}_{\alpha \gamma \phi }$ estimates (Fig. 3). Estimates of nationwide mean log hunting rate per team, $\omega$, followed expectations from how the species were selected; estimates for the commonly hunted red fox and wild boar were higher than estimates for pine marten and beaver. Similarly, the estimates of $\tau$, modelling the variability among counties, were higher for wild boar and beaver, which were selected because their variable distribution.

For $\upsilon$, where a lower value indicates higher intra-HMP variability, red fox was less variable than the other three. No 95% central credibility interval of $\gamma$, which models the association between average harvest rate and intra-HMP variability, overlapped zero. However, the direction of association differed, with the positive estimates of red fox, wild boar, and beaver (Fig. 3E,K,W) indicating that higher average harvest rate was associated with lower variability, whereas pine marten exhibited the reversed trend (Fig. 3Q). Across species, nearly all posterior densities of $\phi$, the effect of hunting team area on harvest rate per area, were located below zero, indicating that harvest rate per area decreases with increased hunting area.

Across all parameters, models, and species, the lowest effective sample size was observed for $\gamma$ of ${\rm M}_{\alpha \gamma }$ for pine marten at 2434.

### Prediction on validation data

**Figure 4:**
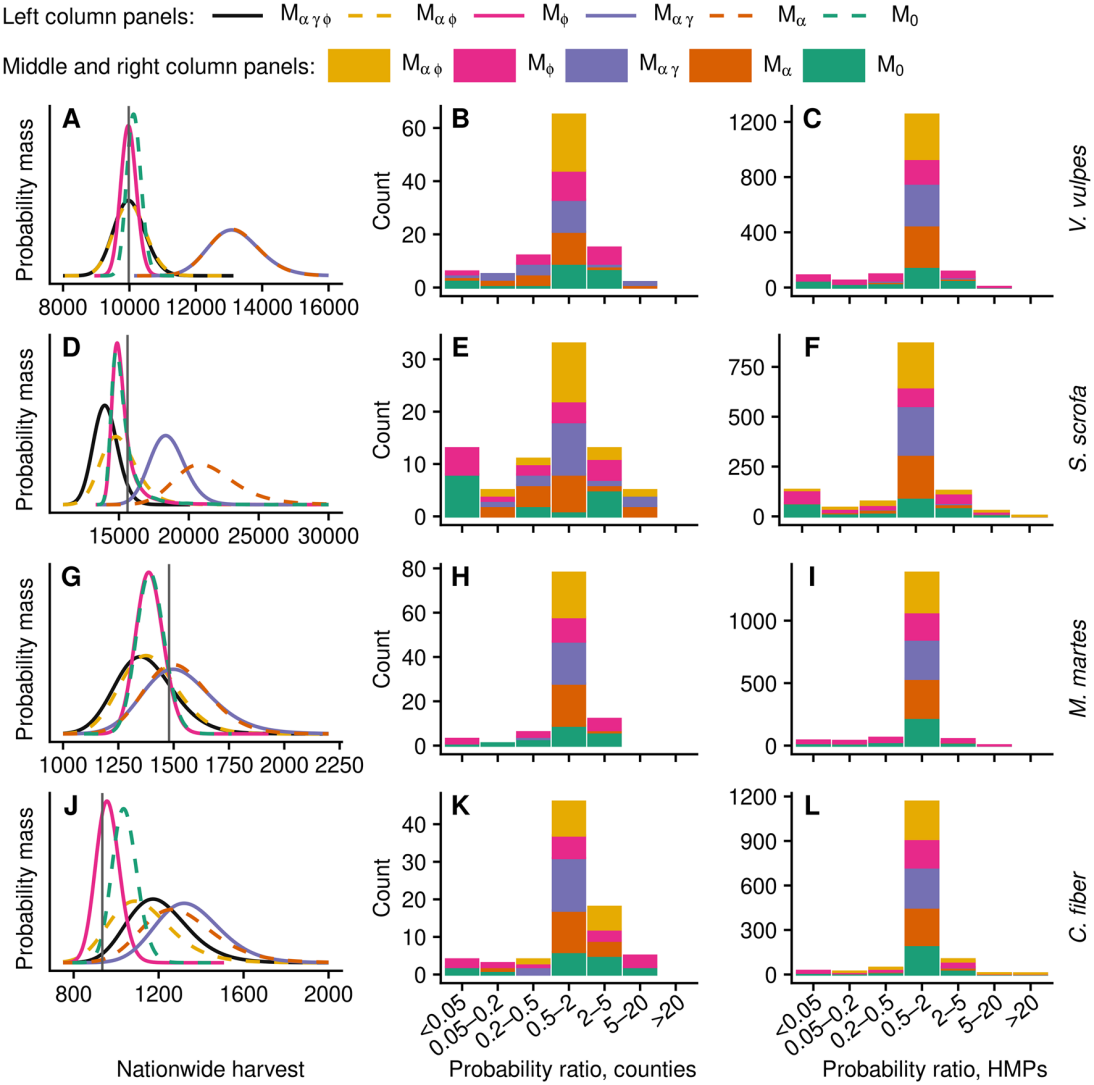
Left column panels: Observed harvest in validation data (vertical grey line) and posterior predictive distributions. Middle and right column panels: ratio of predictive performance of reduced models compared to ${\rm M}_{\alpha \gamma \phi }$ at the county and HMP levels, respectively. Centre bars indicate counties or HMPs where probability mass ratio was within a factor two of the full model, and bars to the left or right indicate worse or better predictive performance, respectively. The figure was generated in R (version 3.6.3, https://www.r-project.org/).

At the nationwide level, PPM is the height at which the distribution curves in the left column panels of Fig. 4 intercept the observed value in the validation set (vertical grey line). Distributions generated with ${\rm M}_{\alpha }$ and ${\rm M}_{\alpha \gamma }$, which account for within-HMP variability but not effect of hunting area on harvest rate per area, overestimates the observed harvest for all species except pine marten.

At county and HMP levels, models ${\rm M}_{\alpha }$, ${\rm M}_{\alpha \gamma }$, and ${\rm M}_{\alpha \phi }$ mostly performed similar to the full model, thus adding to the stacked bars indicating relative predictive performance within the range 0.5–2 in Fig. 4, middle and right column panels. However, the two models excluding within-HMP variability, ${\rm M}_{\phi }$ and ${\rm M}_{0}$, typically performed either better or—much more frequently—worse. The far left stacked bars indicating PPM less than a factor 0.05 times that of ${\rm M}_{\alpha \gamma \phi }$ is dominated by ${\rm M}_{\phi }$ and ${\rm M}_{0}$.

### Leave one out cross validation

For ${\rm M}_{\phi }$ and ${\rm M}_{0}$, the number of observations flagged as PSIS-LOO-CV providing unreliable approximation ranged from 39 (0.8%, ${\rm M}_{\phi }$, beaver) to 153 (3.2%, ${\rm M}_{0}$, wild boar), and these two models contained 661 observation across the four species that would require rerunning with exact LOO-CV. This was deemed too computationally demanding to execute. Thus, we excluded these models from the LOO-CV comparison. For the remaining models, the number of flagged observations ranged from 0 (${\rm M}_{\alpha \gamma \phi }$ and ${\rm M}_{\alpha \gamma }$, wild boar; and ${\rm M}_{\alpha \gamma }$, beaver) to 26 (0.6%, ${\rm M}_{\alpha \phi }$, wild boar).

The full model ${\rm M}_{\alpha \gamma \phi }$ ranked highest for all species (Table 2). However, omitting $\gamma$ as in ${\rm M}_{\alpha \phi }$ only had a pronounced effect for wild boar. For all other species, ELPD difference was within one SE, indicating the average difference in predictive performance was too small relative to the variability across observations to provide definitive conclusions. Omitting $\phi$ as in ${\rm M}_{\alpha \gamma }$ and ${\rm M}_{\alpha }$ showed a distinct decrease in predictive performance for red fox and wild boar, with difference in ELPD above four SE. For pine marten and beaver, ELPD differences were between one and two SE, which is too low to provide definite conclusions.

**Table 2:** Difference in expected log-predictive density (ELPD) relative to the model with best fit for analyses of hunting reports of red fox (*Vulpes vulpes*), wild boar (*Sus scrofa*), pine marten (*Martes martes*), and beaver (*Castor fiber*).

	ELPD difference (SE)			
	${\rm M}_{\alpha \gamma \phi }$	${\rm M}_{\alpha \phi }$	${\rm M}_{\alpha \gamma }$	${\rm M}_{\alpha }$
*V. vulpes*	0 (0)	− 2.9 (7.2)	− 157 (25)	− 153 (25)
*S. scrofa*	0 (0)	− 195 (34)	− 129 (32)	− 337 (39)
*M. martes*	0 (0)	− 5.4 (6.9)	− 9.4 (4.8)	− 16 (10)
*C. fiber*	0 (0)	− 9.7 (10.9)	− 4.1 (3.1)	− 12 (12)

Values in parenthesis indicate the associated standard error (SE) of the estimated difference.

### Estimated harvest

**Figure 5:**
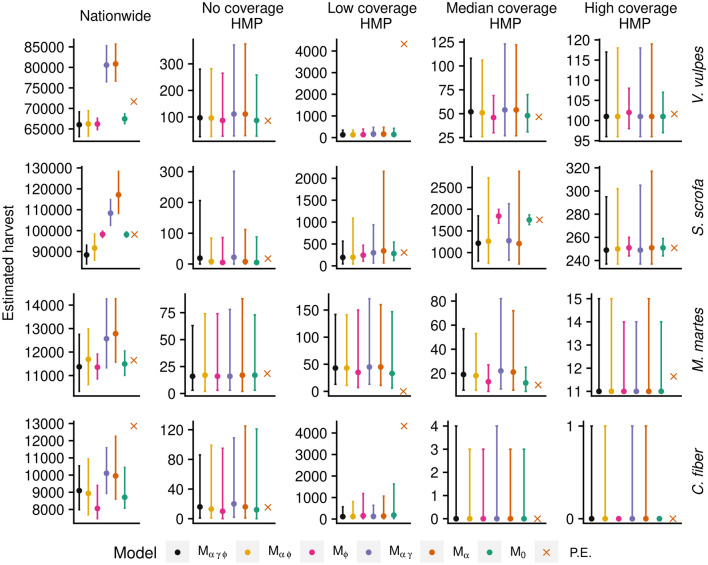
Nationwide and selected HMP total harvest that exemplifies no, low, median, and high coverage in the reports of red fox (*Vulpes vulpes*), wild boar (*Sus scrofa*), pine marten (*Martes martes*), and beaver (*Castor fiber*), predicted with Bayesian models ${\rm M}_{\alpha \gamma \phi } {-}{\rm M}_{0}$ as well as the currently implemented point estimate (P.E.). Error bars indicate 95% posterior predictive central credibility intervals. Location of example HMP are shown in Fig. 6. The figure was generated in R (version 3.6.3, https://www.r-project.org/).

The conclusion that ${\rm M}_{\alpha }$ and ${\rm M}_{\alpha \gamma }$ overestimate nationwide harvest is mirrored in the total harvest estimates of Fig. 5, left column panels. At the HMP level, differences were more elusive due to the large uncertainty represented by 95% credibility intervals, which are particularly wide for HMP with no or low coverage. Models ${\rm M}_{\phi }$ and ${\rm M}_{0}$, which exclude intra-HMP variability, predicts narrower ranges than other models at the nationwide level and for HMP with high coverage, whereas ranges for no, low, and median coverage circuits includes examples of both wider and narrower ranges.

Figure 5 also includes point estimates (P.E.) made with the currently used method. Notable differences were observed, in particular for red fox and beaver. The most extreme differences are found for the low coverage example HMP, which was consistent for all species except wild boar, for which included ranges did not cover this HMP. A closer examination of the HMP reports showed that this HMP had a single reporting hunting team, covering 0.02% of the HMP huntable area. This team reported one red fox and one beaver, which when extrapolating linearly to the entire HMP correspond to more than 4000 culled individuals, which is far above the ranges predicted by any of the HBM. This difference explains most of the discrepancy between P.E. and most of the Bayesian models at the nationwide level. Further, the same low coverage HMP reported zero pine marten, making P.E. estimates zero, which is below the 95% CCI of any of the Bayesian models.

**Figure 6:**
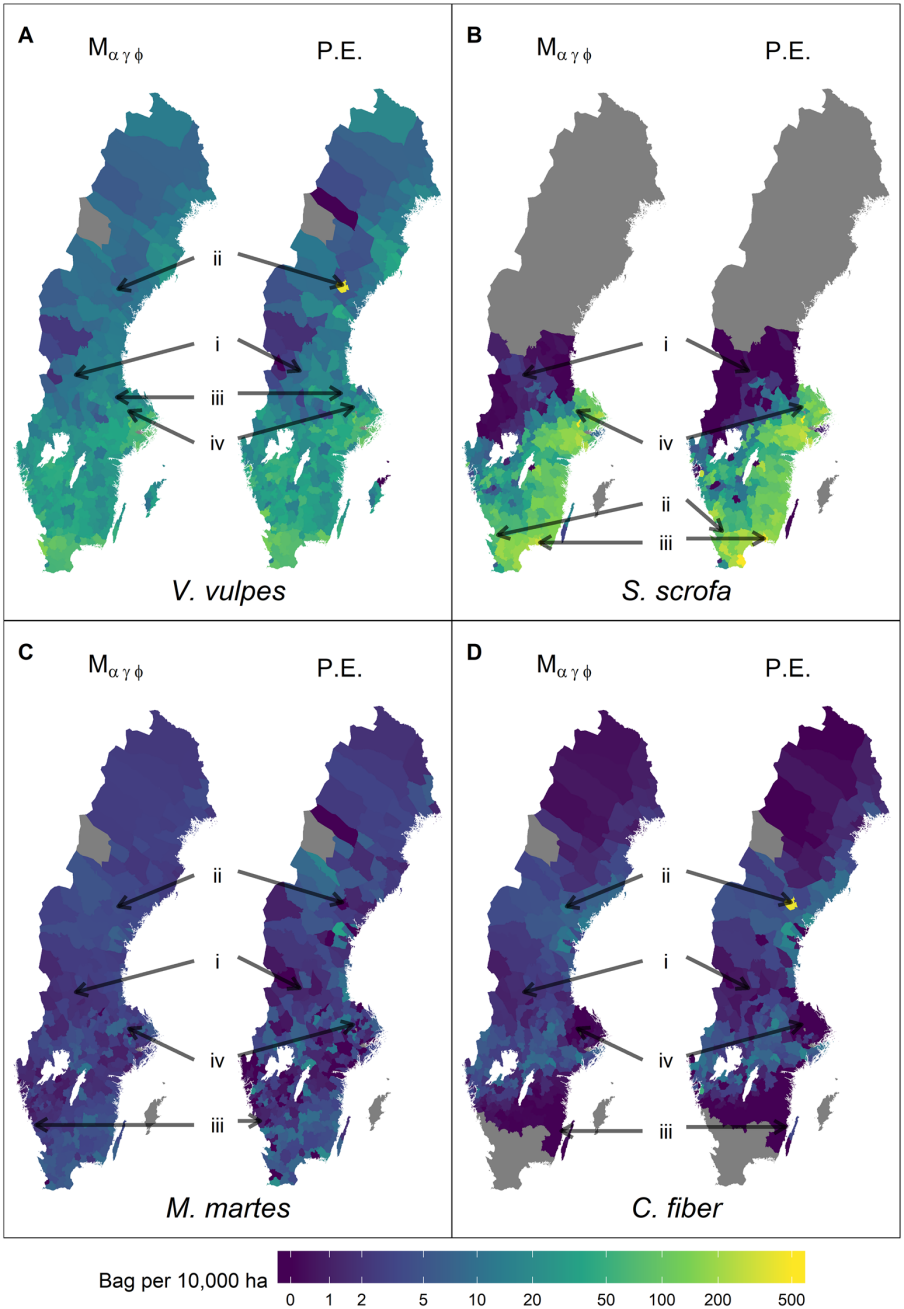
Median estimated harvest per 10,000 ha of model ${\rm M}_{\alpha \gamma \phi }$ (left) and the corresponding point estimate (P.E., right) for red fox (*Vulpes vulpes*), wild boar (*Sus scrofa*), pine marten (*Martes martes*), and beaver (*Castor fiber*). Annotated are HMPs with no (i), low (ii), median (iii), and high (iv) coverage used as examples in Fig. 5. Grey areas indicate HMPs excluded from the analysis because their county had no reported harvest or have no huntable land included in harvest estimation. The figure was generated in R (version 3.6.3, https://www.r-project.org/).

A further comparison is presented in Fig. 6, which compare P.E. harvest to median estimates of ${\rm M}_{\alpha \gamma \phi }$. The overall geographic patterns are similar, but the P.E. exhibits starker contrasts in harvest rates between neighbouring HMP, most notably for the low reporting example HMP of red fox (panel A), pine marten (panel C), and beaver (panel D).

## Discussion

Quantifying hunting harvest is essential for numerous ecological topics, including population estimation^15^, management^1^, and predator–prey interactions^16^, necessitating reliable estimates. We here introduced a novel framework that provides both system specific and general insight.

Using a Poisson model as a starting point (${\rm M}_{0}$), we showed that additional features were interlinked in terms of how they improve estimation. Model ${\rm M}_{0}$ provided good estimates at the national level. However, at HMP and county levels, it underestimated the uncertainty because it assumes equal harvest rate across teams.

The Poisson likelihood is often too restrictive for ecological data, and the Gamma–Poisson/NB distribution is commonly implemented to account for overdispersion^17^. However, while ${\rm M}_{\alpha \gamma }$ and ${\rm M}_{\alpha }$ did approximately as well as ${\rm M}_{\alpha \gamma \phi }$ in terms of predictive density at HMP and county levels, these models induced an overestimation bias, most apparent when aggregating to nationwide harvest. Teams with larger hunting areas harvested less per area (Fig. 3) than teams with smaller areas. At the predictive stage, harvest rates only exhibited by teams with small areas can inaccurately be assigned to teams covering large areas. Models ${\rm M}_{\alpha \gamma \phi }$ and ${\rm M}_{\alpha \phi }$ corrects for this structure and exhibited no bias at the nationwide level.

The importance of association between variability and average harvest rate for predictive ability is debatable. Though the full model gave the highest ELPD for all species, its predictive ability fared substantially better only for wild boar when considering SE of LOO-CV across all observations (Table 2). Further, LOO-CV considers model performance at the lowest level, here individual hunting reports, and even for wild boar, the validation study showed no substantial improvement when considering predictive ability at any level of interest. At the nationwide level, the distributions are similar. In Fig. 4, left column panels, the dashed yellow distribution indicating ${\rm M}_{\alpha \phi }$ even has slightly higher density at the observed harvest (horizontal line) than ${\rm M}_{\alpha \gamma \phi }$ (black) for wild boar and beaver, but differences are too small to draw any definitive conclusions from. At the county and HMP level, the yellow bars indicating the ratio between the predictive densities of and ${\rm M}_{\alpha \phi }$ and ${\rm M}_{\alpha \gamma \phi }$ (Fig. 4, middle and right column panels) shows no apparent trend, with most instances falling within the range 0.5–2.

The hierarchical framework shared by all Bayesian models reduced the sensitivity to low reporting, as exemplified by the difference between the Bayesian models and P.E. in Fig. 5 In fact, no reporting HMPs, which in the current method is informed by the average harvest rate in the county, provide more similar estimates. It may be tempting to simply disregard data from low reporting counties and treat them as having no data. However, what number of reports should then be chosen as cut-off for such approach? The HBM framework offers a continuous alternative. With high reporting, the harvest rate is informed primarily by the HMP specific data. When reporting is low, however, the harvest rate is informed primarily by rates in its county. This “borrowing of strength” is a reason why ecologists have embraced HBM.

It should be noted that an equivalent hierarchical model could be formulated in an ML framework, which would offer the advantage of faster computation. However, while parameter uncertainty estimates can be obtained through e.g. a Hessian matrix or bootstrapping, it is not straightforward to incorporate this uncertainty when models are used for prediction, which is the end goal of the proposed methods. Instead, ML prediction is typically restricted to point estimates of parameters, which can be treacherous in hierarchical models if lower level effects are not clearly separated from zero^18^. Fully Bayesian prediction integrates over parameter uncertainty, here by repeatedly sampling from the joint posterior distribution of all model parameters.

Our analyses also provide insight into hunting practices. Notably, the intra-HMP variability among teams was prominent. Most of the posterior densities of $\upsilon$, which is the logarithm of the shape parameter of the Gamma–Poisson distribution in a HMP of average harvest rate, is below zero for all species (Fig. 3), indicating that the distribution describing intra-HMP variability is more variable than an exponential distribution (the special case of the Gamma distribution with shape one). Thus, even for species considered common game, such as red fox and wild boar, most teams harvest at low rates. Unless this is accounted for in statistical models, estimates of average harvest rate can be biased by the behaviour of individual hunting teams.

Though the importance of $\gamma$ for predictive purposes was found dubious, posterior estimates suggests that the variability among teams was greater in HMP where the average harvest rate was low for red fox, wild boar, and beaver. This behaviour is likely an effect of species distribution. Where the species is at low densities, it is likely not available as game in all areas, especially for range shifting wild boar and beaver. Conversely, the posterior distribution of $\gamma$ for pine marten indicated higher variability among teams where the average harvest rate was high. A plausible explanation is the primary hunting techniques used for pine marten. Though opportunistic rifle hunting occurs, pine marten is primarily hunted through trapping, and large harvest in some areas is likely driven by a small number of specialist hunters with suitable equipment.

The result that teams with access to large areas harvest less per area suggest that harvest rates per team does not scale linearly with hunting area. One explanation is that effort could be invested to obtain a needed harvest, and hunters with small areas could compensate through increased effort. Alternatively, hunters that harvest on smaller areas may have kept their hunting areas small because the productivity of the land is high.

It should be stressed that the presented models are not proposed as the end-all framework for analyses of harvest statistics. We envisage that further extensions to the framework could improve estimation further. However, pragmatism needs to be taken into consideration. With increased model complexity, computational issues become more demanding. Even though Stan is an efficient sampler^10^, tuning of sampler parameters or reparameterizations may be required for complex models, and a one-size-fits-all approach may not be applicable across multiple species (in Sweden currently 49). Tailoring of the framework to individual species may not be feasible under limited resources.

One important caveat of our estimation is that data is not collected randomly, and the introduced modelling approach assumes that reporting rates are independent from harvest rates. It should be noted that reports are submitted jointly for all species in the reporting system, reducing the potential bias that successful or unsuccessful harvesting of a focal species influences whether reporting occurs or not. Yet, we cannot conclude that the voluntary reporting does not impose a bias (see e.g. Aubry et al.^19^). However, assuming that such potential bias does not change abruptly between years, for instance due to changes in hunter efforts, the estimated harvest should still provide a reliable measure of trends. The ability to obtain comparable credibility intervals at HMP, county, and national levels is then essential to differentiate actual changes in harvest rates from randomness in yearly reports. Swedish harvest data have been used to explain shifts in spatial and temporal patterns of the invasive American mink *Mustela vison*^20^, climate effects on mammal populations^21^, and changes in mammal prey species abundance^22,23^.

Carefully designed survey studies targeting all hunters could potentially be used to investigate how reporting hunters differ from the non-reporting population, as has been implemented in other systems^24,25^. A challenge for such a survey study to identify bias in the Swedish system is that harvest reports are made by teams rather than individual hunters, risking incomparable estimates if respondents are selected from the hunter population. The potential presence of a reporting bias does however not reduce the importance of the factors considered here. We argue that HBM, which is apt to integrate multiple sources of information, is an ideal framework to incorporate such survey data, should it become available.

Hunting practises and reporting systems vary among countries. Thus, not all aspects of the introduced framework would be directly transferable. Yet, we believe several key insights from our study should be of relevance across systems. For instance, the high variability in harvest rate within HMPs means that a large proportion of hunters needs to be sampled to capture the population. With low coverage, non-hierarchical approaches can be highly sensitive to individual reports. The HBM framework is well suited to circumvent this, and unlike point estimates, it provides a transparent representation of inherent uncertainty. Further, we identified important structures in the variability among hunters, specifically the negative effect of hunting area on harvest rate per area. We hope these insights will contribute to methodological advances of harvest estimation across systems.

## Supplementary information

Supplementary Information 1.

Supplementary Information 2.
